# Increased Kremen2 predicts worse prognosis in colon cancer

**DOI:** 10.3389/pore.2023.1611082

**Published:** 2023-04-12

**Authors:** Junxian Long, Fengyun Cong, Yousheng Wei, Jungang Liu, Weizhong Tang

**Affiliations:** ^1^ Division of Colorectal and Anal Surgery, Department of Gastrointestinal Surgery, Guangxi Medical University Cancer Hospital, Nanning, Guangxi, China; ^2^ Department of Breast and Thyroid Surgery, The Fifth Affiliated Hospital of Guangxi Medical University, Nanning, Guangxi, China; ^3^ Department of Gastroenteroanal Surgery, The Fifth Affiliated Hospital of Guangxi Medical University, Nanning, Guangxi, China; ^4^ Department of Gynecologic Oncology, Guangxi Medical University Cancer Hospital, Nanning, Guangxi, China; ^5^ Guangxi Clinical Research Center for Colorectal Cancer, Nanning, Guangxi, China

**Keywords:** colon cancer, prognosis, bioinformatic, Kremen2, tumour infiltrating cells

## Abstract

**Background:** Colon cancer (CC) is the fifth most prevalent cancer around the globe and poses a major risk to human health. Even though Kremen2 serves as a prognostic indicator in individuals with malignant tumours, its role in evaluating the prognosis of individuals with colon cancer has not been confirmed.

**Methods:** Here, we examined the protein expression of Kremen2 in CC tissues and paired adjacent normal tissues by immunohistochemistry (IHC), then analyzed the clinical and RNA-seq data presented in The Cancer Genome Atlas (TCGA) database to confirm the relationship between Kremen2 levels and CC. In addition, the associations between Kremen2 mRNA expression and infiltrating immune cells were examined.

**Results:** The study showed that the mRNA expression and protein level of Kremen2 were increased in CC tissues compared with adjacent normal tissues. According to Kaplan–Meier analysis, high Kremen2 expression in CC was linked to poor overall survival and progression-free survival. Clinical correlation analysis highlighted that a high level of Kremen2 expression was strongly linked with tumour progression, particularly lymph node metastasis. Cox regression analysis highlighted that Kremen2 was an independent prognostic indicator for CC. Bioinformatic studies highlighted that Kremen2 might be associated with the immune status in CC.

**Conclusion:** Increased Kremen2 could serve as a potential prognostic CC biomarker.

## Introduction

Colon cancer (CC) is a prevalent malignant tumour, with rising morbidity and ranking fifth globally in cancer-associated mortality (1). According to previous research, in 2020, approximately 1.2 million individuals were diagnosed with CC, and over 570,000 mortalities occurred globally (1). Data from China’s National Central Cancer Registry show that in 2015, there were approximately 376 per 100,000 new cases and 191 per 100,000 cancer deaths because of CC (2). Despite advancements in treatment over the last decades, tumour recurrence is common. There are various causative factors for CC, and the accumulation of genetic and epigenetic changes is the main cause of CC (3). Numerous genetic alterations that occur during colon carcinogenesis have been found by molecular investigations, but the particular genetic alterations that cause CC to develop and grow are yet to be elucidated (4–6). Therefore, discovering additional diagnostic and prognostic biomarkers and therapeutic targets to help improve long-term outcomes for patients with CC is a priority.

Kringle domain-containing protein Kremen was first discovered to have homologous triple-disulphide-linked peptide repeats in the blood coagulation factor prothrombin (7). Mammalian Kremen1 is a dependence receptor that regulates cell death pathways to eliminate aberrant or undesired cells, and Kremen2 is a very potent inhibitor that blocks the homodimerisation of Kremen1 (8). In addition, Kremen1 and Kremen2 are high-affinity secreted protein Dickkopf1 (Dkk1) receptors that work with Dkk1 to inhibit canonical Wnt (Wnt/β-catenin) signalling (9). Two receptor families transduce the Wnt/β-catenin signalling through the β-catenin pathway. By stabilising intracellular β-catenin, frizzled proteins and low-density lipoprotein receptor-related protein (LRP) 5 or LRP6 (LRP5/6) bind Wnts and transmit their signal (10). With Dkk1 and LRP6, Kremen2 forms a ternary complex, inducing rapid endocytosis, and eliminating the Wnt receptor LRP6 from the cellular membrane.

Kremen2 involvement in the development of the embryo, bone, neural ridge, and tumours has been demonstrated by several studies (11, 12). According to The Cancer Genome Atlas (TCGA) data, more than 65% of the tissue samples in 18 different types of cancer, revealed that Kremen2 expression in cancerous tissues was greater in comparison to that in paired normal tissues. This was particularly observed in squamous cell carcinoma of the lung, where the median increase was ten times greater (8). However, Kremen2’s role and expression in CC are yet unclear. In order to understand the regulatory role of Kremen2 in the advancement and metastasis of CC and its link to patients’ pathological characteristics, this study looked at Kremen2 expression in CC. It also laid the foundation for the development of novel therapeutic targets for CC.

## Methodology

### Patients and tissue samples

We collected paraffin specimens from individuals with CC after radical resection at the Department of Gastrointestinal Surgery, Guangxi Medical University Cancer Hospital, between November 2014 and February 2021. In this study, we included 46 adjacent normal tissues and 113 patients with primary CC. The patients underwent radical resection before radiotherapy and chemotherapy, and the diagnosis was established by two pathologists after evaluating the paraffin specimens. The 2019 World Health Organisation Classification of Tumours of the Digestive System was used to guide all pathological diagnoses. Data on survival were collected through telephonic follow-ups. In addition, we collected detailed clinical information, including age, sex, carcinoembryonic antigen (CEA), tumour size, differentiation, peripheral nerve invasion, lymphovascular invasion, deoxyribonucleic acid mismatch repair (MMR), and the American Joint Committee on Cancer tumour node metastasis (TNM) staging system (seventh edition). All participants provided written consent for this research, and this study was approved by the ethics committee of Guangxi Medical University Cancer Hospital (approval number: LW2022123).

### Immunohistochemical (IHC) staining

The sections that had been embedded in paraffin underwent IHC staining. In summary, sections (4 mm thick) were formalin-fixed, paraffin-embedded, regularly deparaffinised, rehydrated, and heated in antigen retrieval citrate buffer (pH 6.0). After that, non-specific antigens were blocked for 20 min with 10% normal goat serum at 37°C and endogenous peroxidase for 20 min with 3% hydrogen peroxide. Subsequently, the rabbit polyclonal antibody anti-KREMEN2 (orb185896; 1:100 dilution; Biorbyt, Cambridge, UK) was incubated at room temperature for 1 h. This was followed by conjugation to the secondary antibody (KIT-5010; Maixin Inc., Fujian, China) and 3, 3′-diaminobenzidine (DAB) (DAB-1031; Maixin Inc., Fujian, China) staining. A primary antibody that had been replaced with phosphate-buffered saline buffer was employed as a negative control.

### IHC staining evaluation

Independent evaluation and scoring of the IHC data were performed by two researchers who were unaware of the clinicopathological characteristics of the individuals. A semi-quantitative scoring method based on intensity scores (InSs) and proportion scores (PSs) was used to assess protein expression. The InSs were stated as follows: 0, no staining; 1, light brown; 2, brown; 3, dark brown. The PSs of stained epithelial cells were as stated: 0% (0), 1%–25% (1), 26%–50% (2), 51%–75% (3), and 76%–100% (4). The formula IS = PS × InS was used to determine the final IHC scores (ISs). Lastly, according to the Kremen2 protein expression, the samples were sorted into high and low expression groups (IS ≥ 8 and IS < 8, respectively).

### Gene expression analysis in the TCGA database

First, we collected gene expression information of different types of tumours in the Tumour IMmune Estimation Resource (TIMER) database (http://timer.cistrome.org/). Further, we downloaded messenger ribonucleic acid sequencing (mRNA-seq) (level 3, HTSeq-Fragments Per Kilobase of transcript per Million mapped reads data) of the colon adenocarcinoma (COAD) cohort and relevant clinical data from TCGA (https://portal.gdc.cancer.gov/). These data were obtained from 30 normal colon tissue samples, 330 colon tumour tissue samples, and 316 patients with CC (basic information, including age, sex, and the TNM stage, while cases without mRNA-seq data and cases with incomplete clinical data and overall survival (OS) of <30 days were excluded). The characteristics of CC patients are shown in Table 1. Perl was used for data processing. Data processing comprised Integration and normalisation of the transcriptome profiling data, the extraction of clinical information, and the merging of the mRNA data.

**TABLE 1:** Characteristics of patients with CC based on TCGA.

Characteristics	Number of cases	Percentages (%)
Sex
Male	172	54.43
Female	144	45.57
Age
≥55	257	81.33
<55	59	18.67
T stage
T1	7	2.22
T2	58	18.35
T3	218	68.99
T4	33	10.44
N stage
N0	191	60.44
N1	72	22.78
N2	53	16.77
M stage
M0	266	84.18
M1	50	15.82

T: tumour; N: node; M: metastasis.

R packages “ggpubr” and “limma” were used to compare Kremen2 mRNA expression between the colon tumour and normal tissue samples (13). On the basis of the levels of Kremen2 mRNA expression in tumour tissues, 316 patients were subsequently enrolled and separated into two groups (the high-expression and low-expression groups). R packages “ggpubr,” “limma,” “survival,” and “survminer” were employed to assess the significance of the Kremen2 mRNA expression level with different pieces of clinical information and the role of Kremen2 in predicting OS (14). Whether the Kremen2 mRNA expression level could independently predict survival in patients with CC was analysed using a multivariate Cox regression model. Our research complied with the TCGA publication guidelines.

### Infiltrating immune cell estimation

The differences between 22 immune cell subtypes in patients with CC belonging to the high- and low-expression groups were analysed with the CIBERSORT method combined with the LM22 feature matrix to estimate the infiltration status of immune cells (15, 16). The association of the Kremen2 mRNA expression level with immune cell infiltration was analysed using the Spearman correlation coefficient.

### Statistical analysis

R software v4.1, Statistical Package for the Social Sciences v21.0, and GraphPad Prism 8 were utilized to perform the statistical analysis. A non-parametric test was employed to determine the variation in Kremen2 protein expression between CC and adjacent normal tissues. The association of the Kremen2 protein expression level with different pieces of clinical information was analysed with the help of Chi-square or Fisher’s exact tests. The Kaplan–Meier (K–M) analysis was employed to assess the role of Kremen2 in predicting the OS. Receiver operating characteristic (ROC) curves were utilised for measuring the prognostic capacity of Kremen2 protein expression. In order to determine if Kremen2 might be employed as an independent prognostic indicator in individuals with CC, univariate and multivariate Cox regression analyses were carried out. *p* < 0.05 was considered a significant value.

## Results

### Kremen2 is overexpressed in CC

**FIGURE 1:**
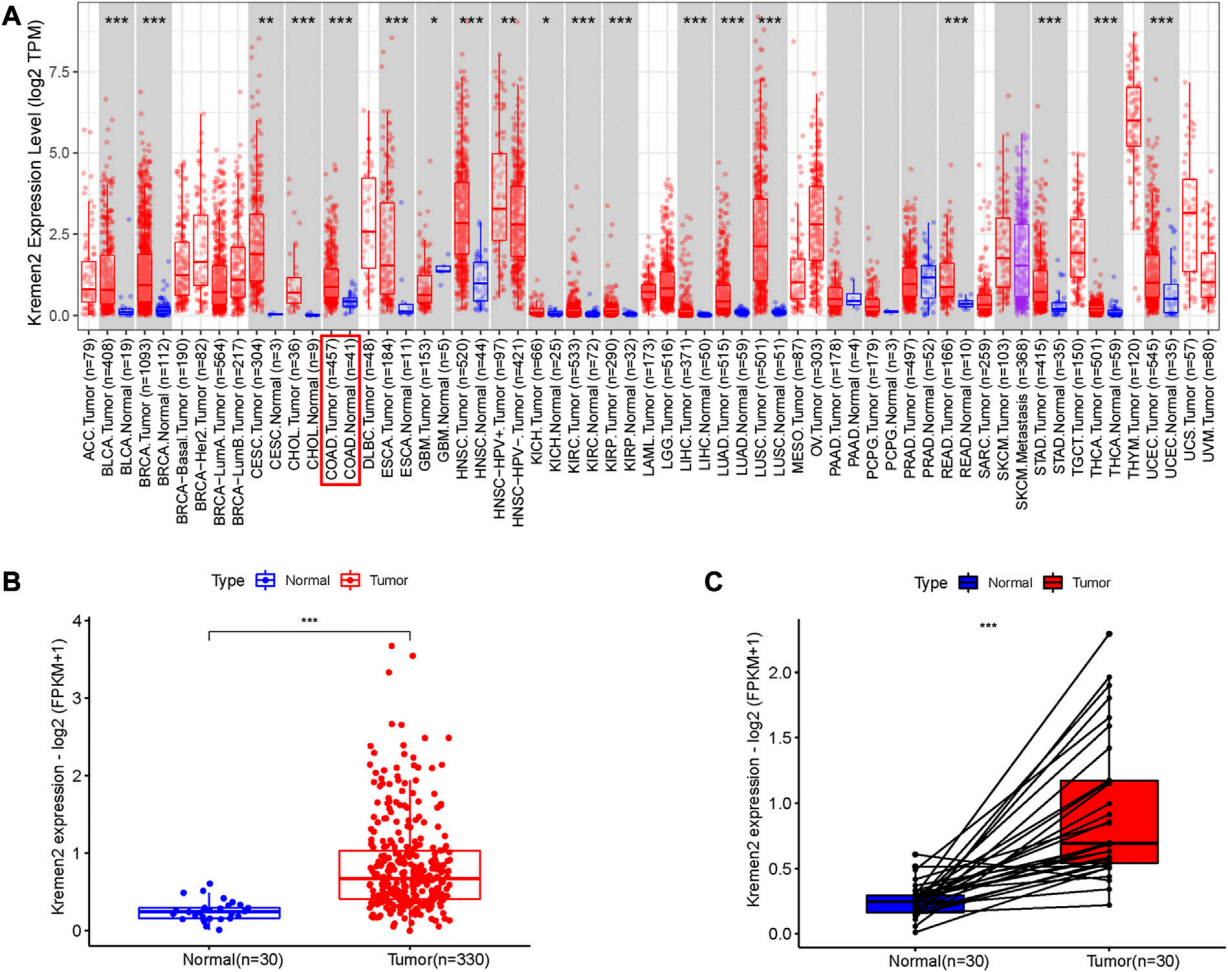
The mRNA expression of Kremen2 in different tumours base on TIMER database and in the COAD base on TCGA database. **(A)** The Kremen2 mRNA expression in different kinds of tumours. **(B)** Unpaired comparison: Kremen2 mRNA expression was elevated in COAD tissues compare to adjacent normal tissues. **(C)** Paired comparison: Kremen2 mRNA expression was elevated in COAD tissues compare to adjacent normal tissues. *: *p* < 0.05, **: *p* < 0.01, ***: *p* < 0.001.

**FIGURE 2:**
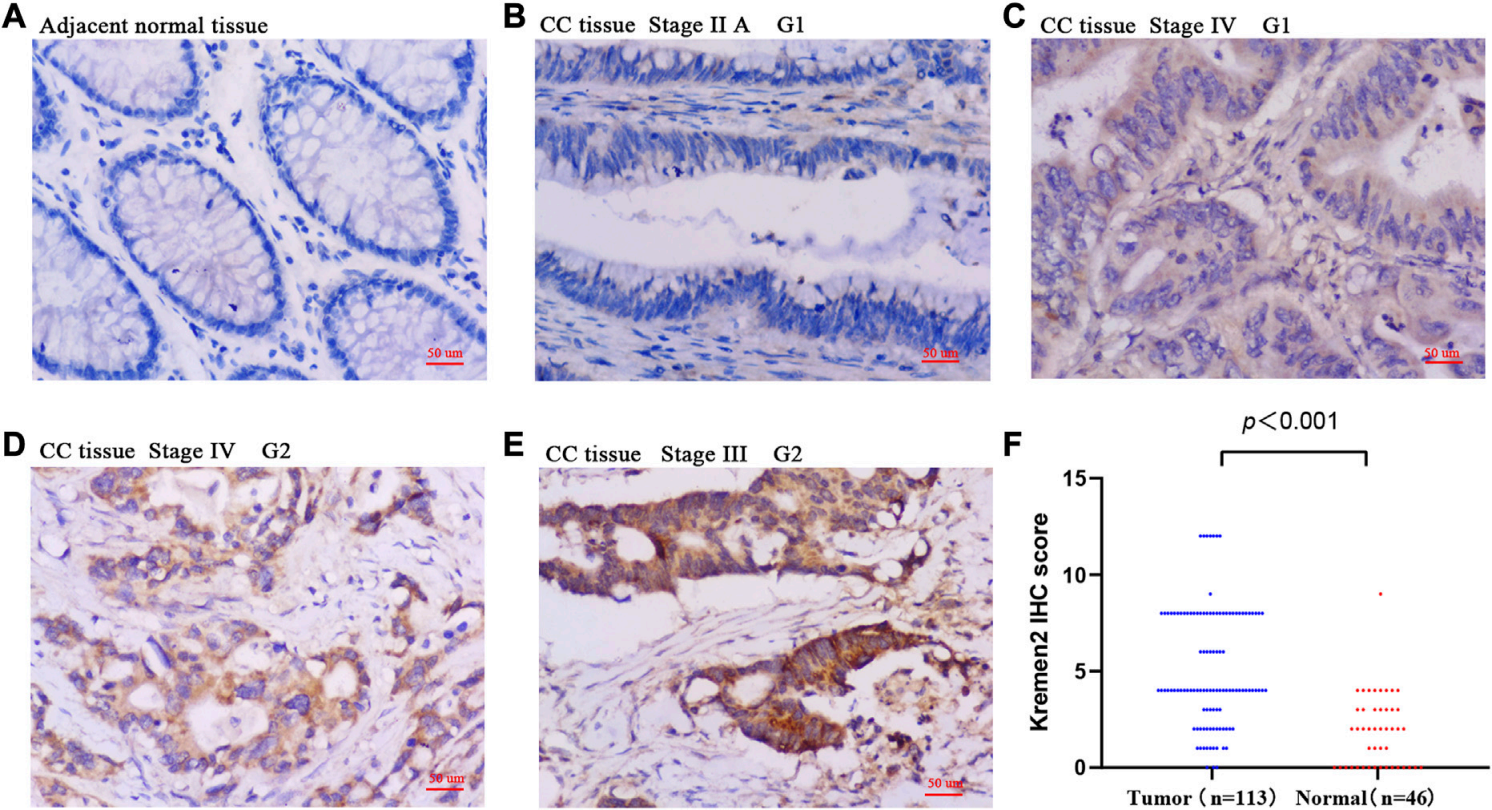
The protein expression of Kremen2 in colon cancer and adjacent normal tissues by using IHC analysis. **(A)** Adjacent normal tissue InS = 0. **(B)** High differentiation (G1), tumour tissue InS = 0. **(C)** High differentiation (G1), tumour tissue InS = 1. **(D)** Moderate differentiation (G2), tumour tissue InS = 2. **(E)** Moderate differentiation (G2), tumour tissue InS = 3. **(F)** The IHC score of Kremen2 protein was elevated in tumour tissues. Magnification ×200, scale bar = 50 μm. InS: intensity scores, IHC: immunohistochemical, CC: colon cancer.

Based on the TIMER data, we observed Kremen2 mRNA overexpression in various tumours, including CC (*p* < 0.001) (Figure 1A). TCGA data analysis revealed that Kremen2 mRNA levels were significantly elevated in CC tissues (Figures 1B, C, *p* < 0.001). We analysed the ISs in 113 tumour tissues and 46 paired adjacent normal tissues to verify Kremen2 protein expression in CC. Using the non-parametric test, we observed that Kremen2 protein expression in CC was remarkably higher in comparison to the adjacent normal tissues (*p* < 0.001) (Figure 2).

### Relationship between Kremen2 protein expression and the clinicopathological features of individuals with CC

Subgroup analysis was performed based on the age, sex, tumour site, TNM stage, CEA, tumour size, differentiation, peripheral nerve invasion, lymphovascular invasion, and MMR for determining the association of Kremen2 protein expression and clinicopathological parameters. In contrast with individuals with CC having no lymph node metastasis, individuals with CC having lymph node metastasis had substantially greater levels of Kremen2 protein expression (*p* = 0.036). In addition, we observed that Kremen2 protein correlated with sex and age in patients with CC. Female patients (*p* = 0.025) and patients aged <55 years (*p* = 0.013) tended to have a high Kremen2 protein expression. However, there was no considerable association between Kremen2 protein expression in CC and other clinicopathological parameters (*p* > 0.05) (Table 2).

**TABLE 2:** The correlation between Kremen2 protein expression and clinicopathological parameters of CC.

Parameters	Kremen2	
	High	Low	*p*
Sex			0.025
Male	20	52	
Female	20	21	
Age			0.013
<55	21	21	
≥55	19	52	
Tumour size (cm)			0.438
≤5	26	42	
>5	14	31	
T stage			0.221
Tis/T1/T2	1	8	
T3/T4	39	65	
N stage			0.036
N0	17	46	
N1/N2	23	27	
M stage			0.129
M0	32	67	
M1	8	6	
Differentiation			0.163
High/Moderate	29	61	
Poorly	11	12	
Peripheral nerve invasion			0.547
Positive	21	34	
Negative	19	39	
Lymphovascular invasion			0.890
Positive	12	21	
Negative	28	52	
CEA (ng/mL)			0.362
≤5	20	43	
>5	20	30	
MMR			0.708
DMMR	5	11	
PMMR	35	62	

MMR: DNA mismatch repair; dMMR: defective DNA mismatch repair; pMMR: proficient DNA mismatch repair.

### The relationship between Kremen2 mRNA expression and clinical properties

**FIGURE 3:**
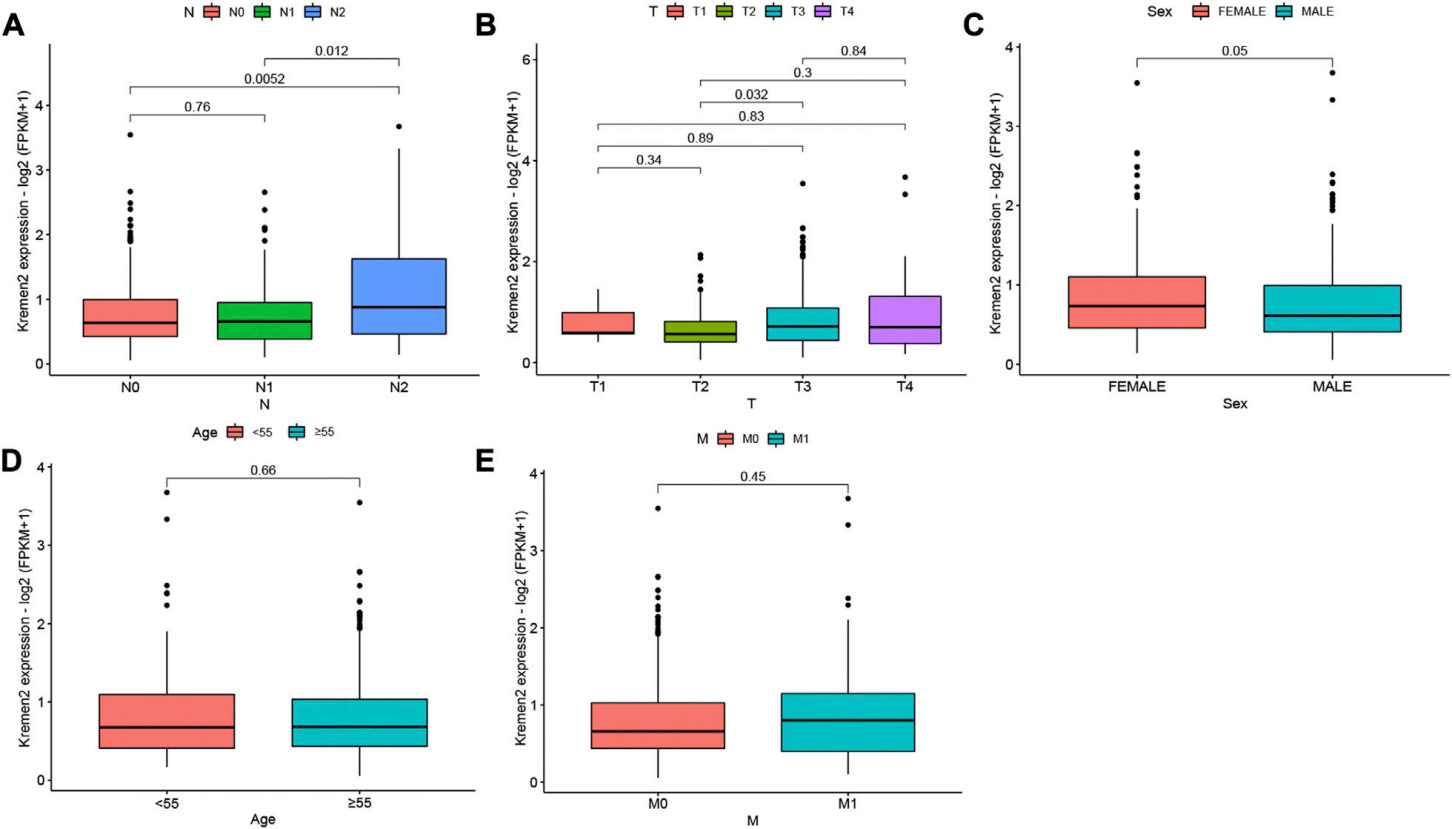
Relationship between Kremen2 mRNA expression and clinicopathological characteristics of CC patients based on TCGA. Box plot showing the relation of Kremen2 mRNA to **(A)** N stage, **(B)** T stage, **(C)** sex, **(D)** age and **(E)** M stage.

When we examined the relationship between clinicopathological parameters and Kremen2 mRNA expression in patients with CC based on TCGA, we observed that high Kremen2 mRNA expression was linked to tumour N stage (from N0 to N2 and from N1 to N2) (*p* = 0.005, 0.012) (Figure 3A). In terms of the T stage, progression from T2 to T3 was correlated with elevated Kremen2 mRNA expression levels (*p* = 0.032) (Figure 3B). However, Kremen2 mRNA expression was not related to sex, age, or the M stage (Figures 3C–E).

### High Kremen2 expression in CC was linked to a poor prognosis

**FIGURE 4:**
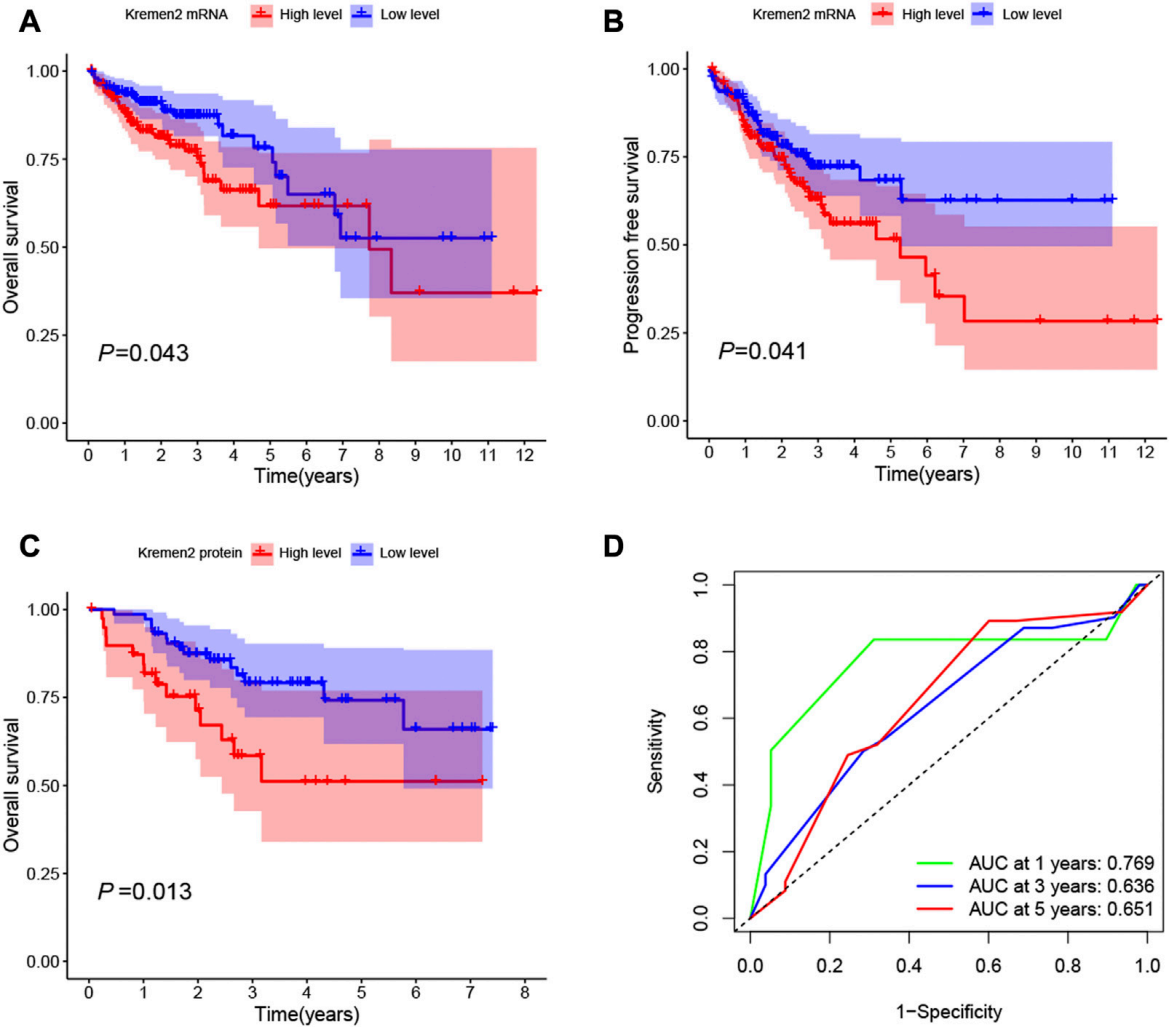
Kaplan-Meier survival curves of CC patients with high and low Kremen2 expression levels. **(A)** OS in CC patients with differential Kremen2 mRNA expression. **(B)** Progression free survival in CC patients with differential Kremen2 mRNA expression. **(C)** OS in CC patients with differential Kremen2 protein expression. **(D)** The AUC score at 1, 3, 5 years in Kremen2 protein expression. AUC, area under the ROC curve.

The K–M survival curve was implemented to determine the association of Kremen2 expression and survival in CC. As per the K–M chart, CC cases with elevated Kremen2 mRNA expression had a shorter OS (TCGA, *p* = 0.043) (Figure 4A) and progression-free survival (TCGA, *p* = 0.041) (Figure 4B). The K–M survival curve also revealed that high Kremen2 protein expression was linked to a poor prognosis (IHC, *p* = 0.013) (Figure 4C). Subsequently, the ROC curve analysis was employed to analyse the predictive value of Kremen2 protein expression, promising a prognosis value of the signature for CC over survival. The area under the curve scores over 1, 3, and 5 years (0.769, 0.636, and 0.651, respectively), were observed on the ROC curves (Figure 4D).

### Kremen2 protein expression is an independent prognostic factor for patients with CC

**FIGURE 5:**
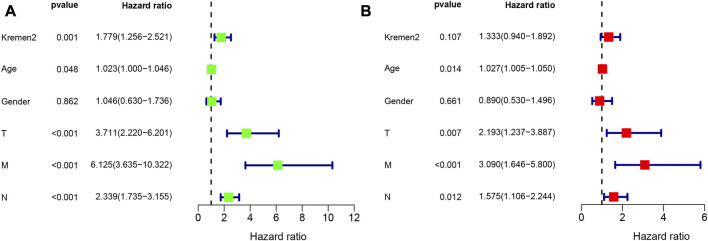
Univariate and multivariate Cox regression analyses of OS for CC patients in the TCGA. **(A)** Univariate Cox analyses. **(B)** Multivariate Cox analyses.

Using clinical data from TCGA, we observed that age (*p* = 0.014), the T stage (*p* = 0.007), the N stage (*p* = 0.012), and the M stage (*p* < 0.001) were independent risk factors for the prognosis of individuals with CC (Figure 5). As per the univariate analysis results, high Kremen2 mRNA expression was strongly related to poor prognosis (*p* = 0.001), while it was not correlated in the multivariate analysis (*p* = 0.107). In addition, the univariate Cox regression analysis revealed a substantial correlation between high Kremen2 protein expression and a poor prognosis (*p* = 0.017). Other clinical parameters, including the N stage (*p* = 0.009), the M stage (*p* = 0.000), and peripheral nerve invasion (*p* = 0.018), were linked to a poor prognosis (Table 3). Furthermore, the multivariate analysis revealed that high Kremen2 protein expression (*p* = 0.036) and the M stage (*p* = 0.001) were independently linked with a poor prognosis (Table 3).

**TABLE 3:** Univariate and multivariate Cox regression analyses of OS for CC patients in the IHC cohort.

Parameters	Univariate analysis			Multivariate analysis		
	*P*	HR	95% CI	*P*	HR	95% CI
Sex (Male vs. Female)	0.158	0.590	0.283–1.228			
Age (≥55 vs. < 55)	0.426	1.378	0.626–3.032			
T stage (T3/T4 vs. Tis/T1/T2)	0.246	3.263	0.442–24.065			
N stage (N1/N2 vs. N0)	0.009	2.742	1.283–5.858	0.698	1.202	0.475–3.038
M stage (M1 vs. M0)	0.000	6.354	2.851–14.161	0.001	4.942	1.945–12.555
CEA (ng/mL) (>5 vs. ≤5)	0.077	1.943	0.930–4.060			
Tumour size (cm) (>5 vs. ≤5)	0.699	0.860	0.400–1.850			
Differentiation (High/Moderate vs. Poorly)	0.396	0.701	0.309–1.590			
Peripheral nerve invasion (Positive vs. Negative)	0.018	2.605	1.182–5.738	0.089	2.057	0.895–4.728
Lymphovascular invasion (Positive vs. Negative)	0.090	1.888	0.906–3.936			
Kremen2 protein (High vs. Low)	0.017	2.446	1.177–5.084	0.036	2.231	1.052–4.730
MMR (dMMR vs. pMMR)	0.202	0.392	0.093–1.653			

### Correlation between Kremen2 mRNA levels and immune cell infiltration levels

**FIGURE 6:**
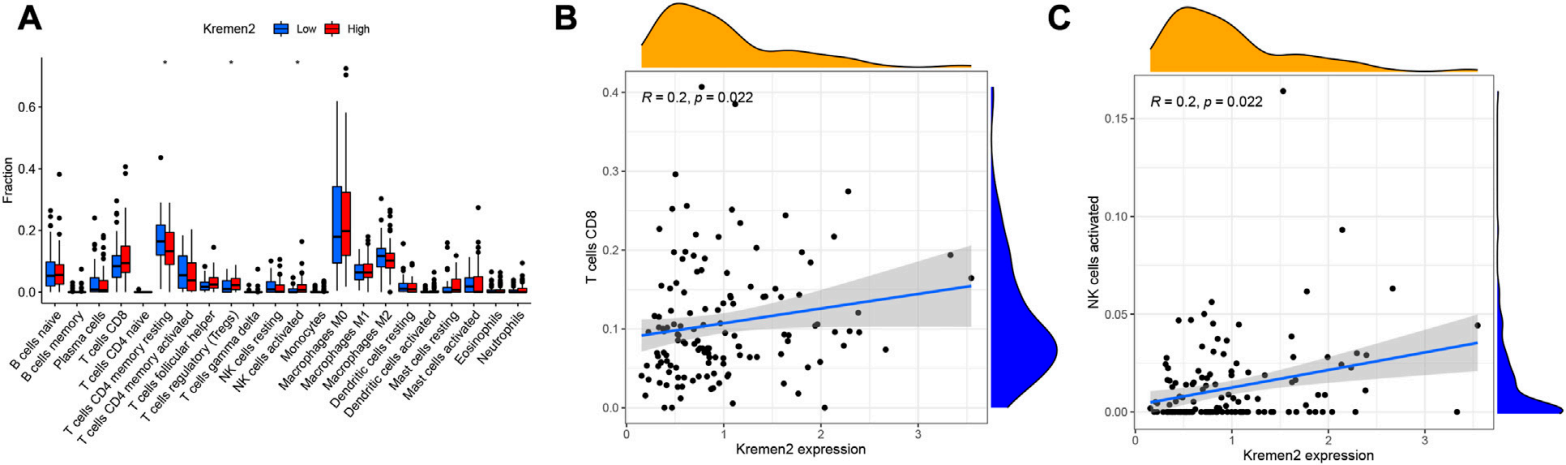
Association analysis between the Kremen2 mRNA levels and immune cells infiltration levels. **(A)** The immune cells between high-expression and low-expression groups; *p < 0.05. **(B)** The correlation between CD8 T cells and Kremen2 mRNA level. **(C)** The correlation between activated NK cells and Kremen2 mRNA level.

Tumour-infiltrating lymphocytes can independently reflect cancer survival (17). Herein, we assessed the link between Kremen2 mRNA expression and the infiltration of 22 immune cell subtypes in TCGA-COAD. Regulatory T cells (Tregs) (*p* = 0.034) and activated natural killer (NK) cells (*p* = 0.020) were the immune cells with a substantially higher infiltration level in the high-expression group. CD4^+^ memory resting T cell infiltration level was substantially higher in the low-expression group in comparison to that in the high-expression group (*p* = 0.031) (Figure 6A). According to the correlation analyses, CD8^+^ T cell and activated NK cell infiltration were positively linked to Kremen2 mRNA expression (Figures 6B, C, r = 0.2, *p* = 0.022; r = 0.2, *p* = 0.022).

## Discussion

CC is among the most common cancers globally, and its treatment and rehabilitation cause significant pain to patients (18). Despite advancements in diagnostic and treatment modalities, judging the prognosis of individuals with CC needs further research (19, 20). Therefore, it is essential to further define the underlying mechanism of CC, particularly in terms of the prognostic molecular markers. Currently, there is growing interest in studying the mechanism of Kremen2 in tumorigenesis and tumour development.

Kremen2 plays a crucial role in neural crest induction (11), bone formation (12, 21), and tumorigenesis (8, 22). Kremen2 inhibits cell apoptosis by inhibiting homodimerisation of Kremen1. Conversely, Kremen2 functions as a receptor of Dkk1, forming a trimer with LRP5/6 protein to internalise LRP5/6, and block the Wnt-LRP5/6-frizzled complex formation, thus enhancing the Dkk1-mediated suppression of the Wnt/β-catenin signalling pathway. Another research discovered that Kremen2 might bind to LRP5/6 and sustain its presence on the cellular membrane in the absence of the Dkk1 protein, activating the Wnt/β-catenin signalling pathway (23). In conclusion, Kremen2 exhibits biological effects in a variety of biological environments. Recent research reveals that Kremen2 is upregulated in various tumours, such as renal clear cell carcinoma, breast invasive carcinoma, COAD, and stomach adenocarcinoma, in comparison to that in normal tissues (8). Dkk1 and Kremen expression in combination may act as indicators of the osteoblastic response to breast and prostate cancer bone metastases in cancer cells (24). The proliferation of gastric cancer cells was considerably inhibited after knocking down Kremen2, indicating that Kremen2 may exert oncogene-like functions, thus promoting gastric cancer proliferation (25).

However, the role and expression of Kremen2 in CC are yet unclear. In the present investigation, using TCGA-COAD, we observed that Kremen2 mRNA expression was substantially higher in CC tissues in comparison to that in normal tissues. Subsequently, the IHC results also revealed that Kremen2 protein was upregulated in CC tissues, suggesting Kremen2’s involvement in CC carcinogenesis. Prognostic data from TCGA-COAD and the IHC cohort revealed that high Kremen2 expression was strongly linked to poor prognosis in patients regardless of the mRNA or protein levels. This further confirmed the possibility of Kremen2 serving as a prognostic molecule for CC.

Lymph node staging is crucial for determining the prognosis and therapy stratification in CC (26, 27). Lymph node metastasis is associated with an adverse clinical course and is an indication of adjuvant chemotherapy (28). According to the clinicopathological data-based correlation study, Kremen2 mRNA expression was strongly linked to the N stage. Using the IHC cohort, we then verified the association of Kremen2 protein expression with the clinicopathological data and observed that elevated Kremen2 protein expression levels also predicted a poor N stage. The relatively higher Kremen2 expression in the advanced N stage indicated that it could be linked to tumour progression in CC.

According to the univariate and multivariate Cox regression analyses, Kremen2 protein expression can serve as an independent prognostic factor for individuals with CC, the same as the M stage. Consequently, we believed that Kremen2 could serve as a reference for prognostic prediction in individuals with CC. However, further research and clarification regarding the specific mechanism *via* which Kremen2 functions is warranted.

Increasing evidence suggests that malignant tumours rely on tumour cells and the tumour microenvironment, such as extracellular matrix molecules, inflammatory mediators, and immune cells (29, 30). Tumour-infiltrating immune cells are valuable for CC diagnosis and progression and prognosis determination (31–33). Prior research has highlighted that the efficacy of immunotherapy decreases because of the antitumor immune effects of Tregs, thereby promoting immune evasion and cancer progression (34). Therefore, anti-Treg immunotherapy may be highly effective in individuals with CC. NK cells have been reported as natural lymphocytes that may eliminate cancer cells or viral infections (35). Imai et al. (36) observed that tumour progression was linked to a reduced killing capacity of NK cells in peripheral blood. As a result, NK cells are promising targets for anti-CC immunotherapy because they control tumorigenesis. The individuals with CC having a higher proportion of CD4^+^ memory resting T cells and activated NK cells had a better prognosis. On the other hand, a lower proportion of CD8^+^ T cells had a worse prognosis (37). Herein, we observed that Tregs and activated NK cell infiltration levels were significantly higher in the Kremen2 mRNA high-expression group, with attenuated CD4^+^ memory resting T cell infiltration. According to the correlation analysis, CD8^+^ T cell and activated NK cell infiltration were positively linked with Kremen2 mRNA expression, but the association is weak. These results imply that Kremen2 may be associated with the immune status in CC. However, more studies on the association between Kremen2 and CC immune infiltration are warranted.

## Conclusion

**FIGURE 7:**
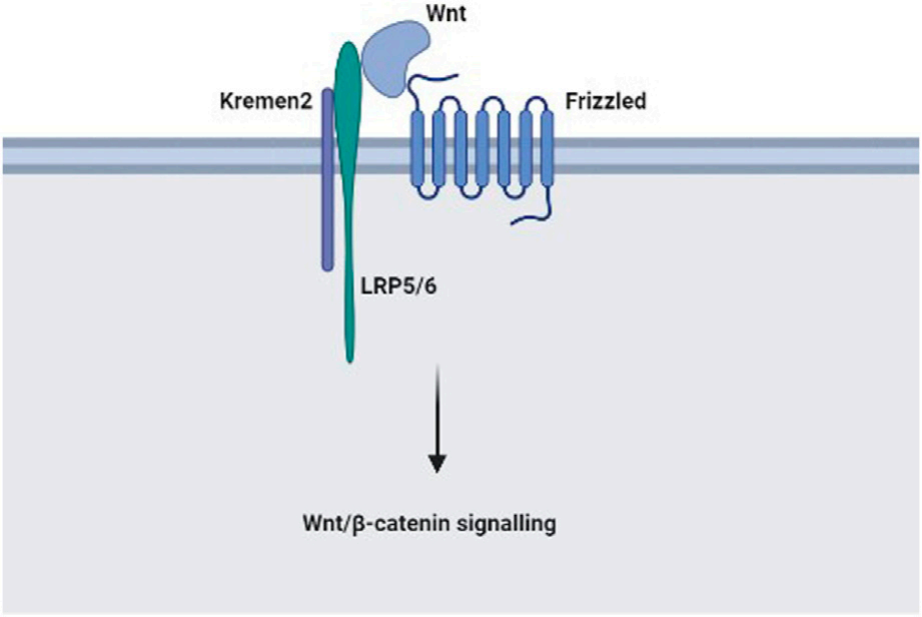
The possible mechanism of Kremen2 in colon cancer disease progression: In the absence of Dkk1, Kremen2 activates the Wnt/β-catenin signalling pathway by maintaining LRP5 or LRP6 at the plasma membrane.

In summary, this research highlighted that Kremen2 expression was upregulated in CC tissues and was strongly linked to tumour progression and prognosis in individuals with CC. This shows that Kremen2 could be a new CC biomarker. Furthermore, a high Kremen2 expression level might predict a poor prognosis, and Kremen2 protein expression level could serve as an independent prognostic factor. Furthermore, Kremen2 may be associated with the immune status in CC. The specific mechanism of Kremen2 in CC disease progression is still unclear. According to relevant literature and this study, Kremen2 may be involved in CC disease progression by activating the Wnt/β-catenin signalling pathway, and the possible mechanism is shown in Figure 7. However, our results are only based on bioinformatic analysis and tissue validation, and further *in vitro* as well as *in vivo* studies are warranted to investigate the biological relationship and function of Kremen2.

## Data Availability

The original contributions presented in the study are included in the article/supplementary material, further inquiries can be directed to the corresponding author.
